# Enhancing Precision and Efficiency in Knee Arthroplasty: A Comparative Analysis of Computer-Assisted Measurements with a Novel Software Tool versus Manual Measurements for Lower Leg Geometry

**DOI:** 10.3390/jcm12247581

**Published:** 2023-12-08

**Authors:** Ulrike Wittig, Amir Koutp, Patrick Reinbacher, Konstanze Hütter, Andreas Leithner, Patrick Sadoghi

**Affiliations:** 1 Department of Orthopaedics and Trauma, Medical University of Graz, 8036 Graz, Austria; ulrike.wittig@medunigraz.at (U.W.); patrick.reinbacher@medunigraz.at (P.R.); konstanze.huetter@medunigraz.at (K.H.); andreas.leithner@medunigraz.at (A.L.); patrick.sadoghi@medunigraz.at (P.S.); 2 Department of Trauma Surgery, Landesklinikum Wiener Neustadt, 2700 Wiener Neustadt, Austria

**Keywords:** leg axis, leg axis angles, leg axis alignment, planning software

## Abstract

(1) Background: The aim of this prospective study was to evaluate measurement software in comparison with manual measurements using inter-observer and intra-observer variability on radiographs in the preoperative planning of total knee arthroplasty. (2) Methods: Two independent observers retrospectively measured the mechanical lateral proximal femoral angle (mLPFA), the mechanical lateral distal femoral angle (mLDFA), the joint line convergence angle (JLCA), the mechanical medial proximal tibial angle (mMPTA), the mechanical lateral distal tibial angle (mLDTA), the hip–knee angle or mechanical tibial–femoral axis angle (HKA), and the anatomical–mechanical angle (AMA) on 55 long-leg anteroposterior radiographs manually twice, followed by measurements using dedicated software. Variability between manual and computer-aided planning was assessed, and all measurements were performed a second time after 14 days in order to assess intra-observer variability. (3) Results: Concerning intra-observer variability, no statistically significant difference was observed regarding the software-based measurements. However, significant differences were noted concerning intra-observer variability when measuring the mLDFA and AMA manually. Testing for statistical significance regarding variability between manual and software-based measurements showed that the values varied strongly between manual and computer-aided measurements. Statistically significant differences were detected for mLPFA, mLDFA, mMPTA, and mLPTA on day 1, and mLPFA, mMPTA, and mLPTA on day 15, respectively. (4) Conclusions: Preoperative planning of leg axis angles and alignment using planning software showed less inter- and intra-observer variability in contrast to manual measurements, and results differed with respect to manual planning. We believe that the planning software is more reliable and faster, and we would recommend its use in clinical settings.

## 1. Introduction

While revision total knee arthroplasty (TKA) is not commonly associated with the surgical technique, it remains crucial to recognize its significance, as factors like inappropriate component size, malposition, and malalignment of the components can still contribute to the necessity for revision [1,2]. Therefore, thorough preoperative planning of relevant leg axis angles and, thus, varus/valgus alignment is an important factor in order to achieve optimal postoperative knee function [3].

Thus, preoperative planning is an important tool in total knee arthroplasty (TKA), especially concerning a reduction in intraoperative errors related to implant sizing, soft tissue balancing, and bony resections [4,5]. Consequently, many researchers have aimed to increase the reliability of preoperative planning [6,7,8].

The international gold standard in clinical preoperative planning is based on two-dimensional (2D) geometrical analysis of anterior–posterior (AP) standing long-leg radiographs by placing translucent templates on the radiographs [7,9]. Several studies have proposed that digital 2D planning might be more precise regarding the prediction of implant size [10,11]. Moreover, some studies have evaluated the accuracy of computed tomography (CT)-based three-dimensional (3D) planning and suggested that this might be more precise concerning the alignment and rotation of the components [12,13,14]. However, the accuracy of the novel measurement software Image Biopsy Lab (Vienna, Austria) has not been described on long-leg radiographs.

The aim of this prospective study was, therefore, to evaluate the above-mentioned measurement software in comparison with conventional manual measurements using inter-observer and intra-observer variability on 2D radiographs.

Our hypothesis is that the use of measurement software is more reliable and efficient in preoperative planning compared to manual measurements.

## 2. Materials and Methods

**Figure 1:**
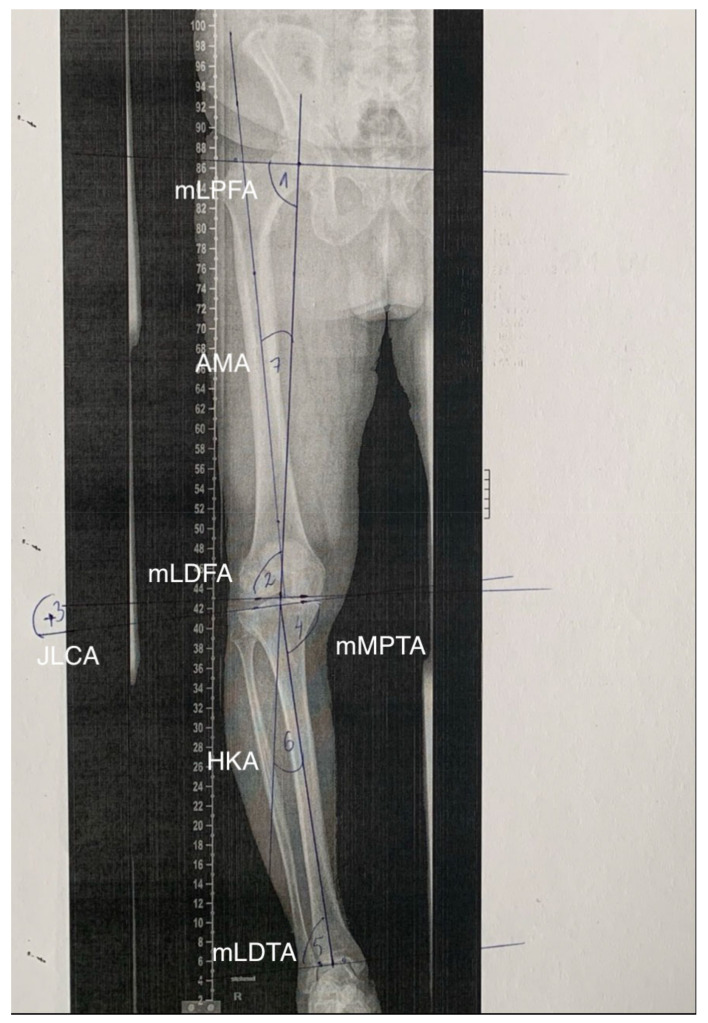
A graphical depiction of the angles measured on a long-leg radiographic view. (1) mLPFA; (2) mLDFA; (3) JLCA; (4) mMPTA; (5) mLDTA; (6) HKA; (7) AMA.

This study was approved by the institutional review board (blinded for review). Fifty-five pseudonymized standardized anteroposterior long-leg views were randomly selected from a patient collective consisting of surgical candidates for total knee arthroplasty between January 2021 and April 2021, as these radiographs were taken for preoperative planning [9,10]. The radiographs were independently reviewed by two observers, first manually and then using measurement software LAMA^TM^ (Image Biopsy Lab GmbH, Vienna, Austria). The measurement was carried out by two senior residents with experience and specialization in knee arthroplasty. In a second step, two independent reviewers were selected to evaluate the variability between manual and computer-aided planning, which was defined as inter-observer variability. Moreover, the angles were then measured a second time after a time interval of 14 days to assess intra-observer variability. Seven standardized angles were measured in degrees as follows: (1) the mechanical lateral proximal femoral angle (mLPFA); (2) the mechanical lateral distal femoral angle (mLDFA); (3) the joint line convergence angle (JLCA); (4) the mechanical medial proximal tibial angle (mMPTA); (5) the mechanical lateral distal tibial angle (mLDTA); (6) the hip–knee angle or mechanical tibial–femoral axis angle (HKA); and (7) the anatomical–mechanical angle as the angle between the anatomical and mechanical axis of the femur (AMA). A graphical depiction of these angles on a long-leg view is presented in Figure 1.

### 2.1. Manual Measurements

The manual measurements were performed on blinded prints of the radiographs by drawing the baselines through significant points of the proximal and distal femur and tibia, respectively. The first baseline was drawn through the central point of the femoral head and the tangent of the great trochanter. The second baseline runs through the most prominent protrusions of the medial and lateral femoral condyles. Analogously, the most prominent points of the proximal and distal tibia were connected with the third and fourth baseline. Next, the anatomical axis was drawn by connecting two central lines through the diaphyses of the femur and tibia. Additionally, the mechanical axis of the femur was drawn through the central point of the femoral head and the center of the femoral condyles. The mechanical axis of the tibia was drawn through the center of the tibial tubercles and the center of the previously marked distal tibial baseline. The mLPFA is the lateral angle between the femoral mechanical axis and the first baseline. The mLDFA is the lateral angle between the femoral mechanical axis and the second baseline. The JCLA, which has a positive value in case of varus deformity and a negative value in case of valgus deformity, constitutes the angle between the second and third baseline. The mMPTA is the medial angle between the third baseline and the mechanical axis of the tibia. The mLDTA is defined as the lateral angle between the fourth baseline and the mechanical axis of the tibia. The HKA, whose positive or negative value is determined analogously to the JLCA, constitutes the angle between the mechanical femoral and mechanical tibial axis. Finally, AMA is the angle between the anatomical and mechanical femoral axis.

### 2.2. Software Measurements

**Figure 2:**
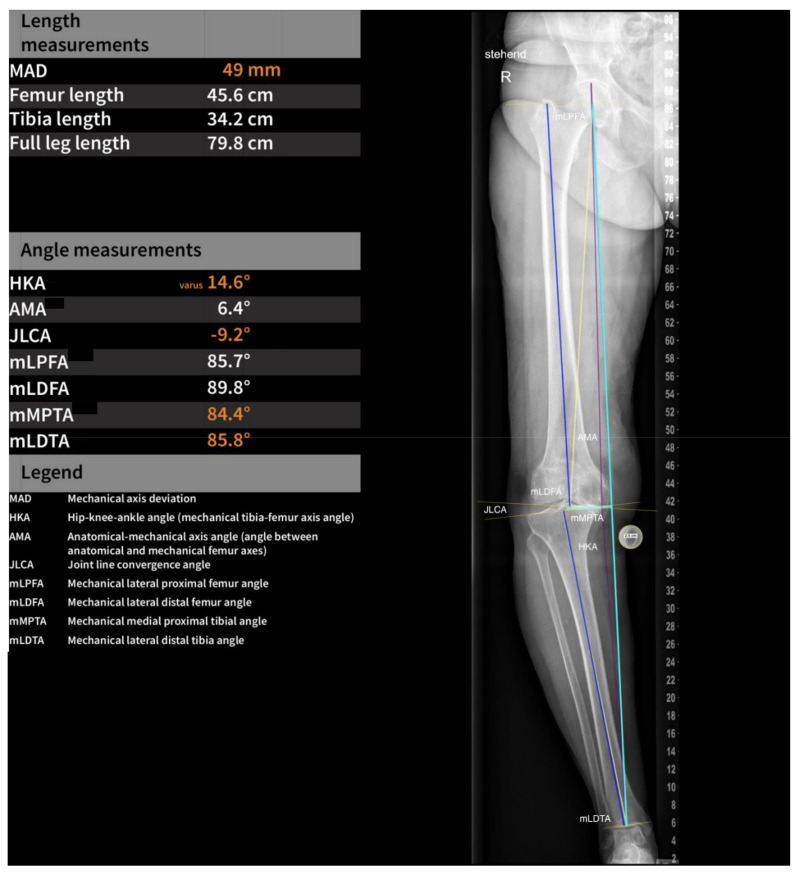
A graphical depiction of software-based measurements of standardized angles on a longleg view.

**Figure 3:**
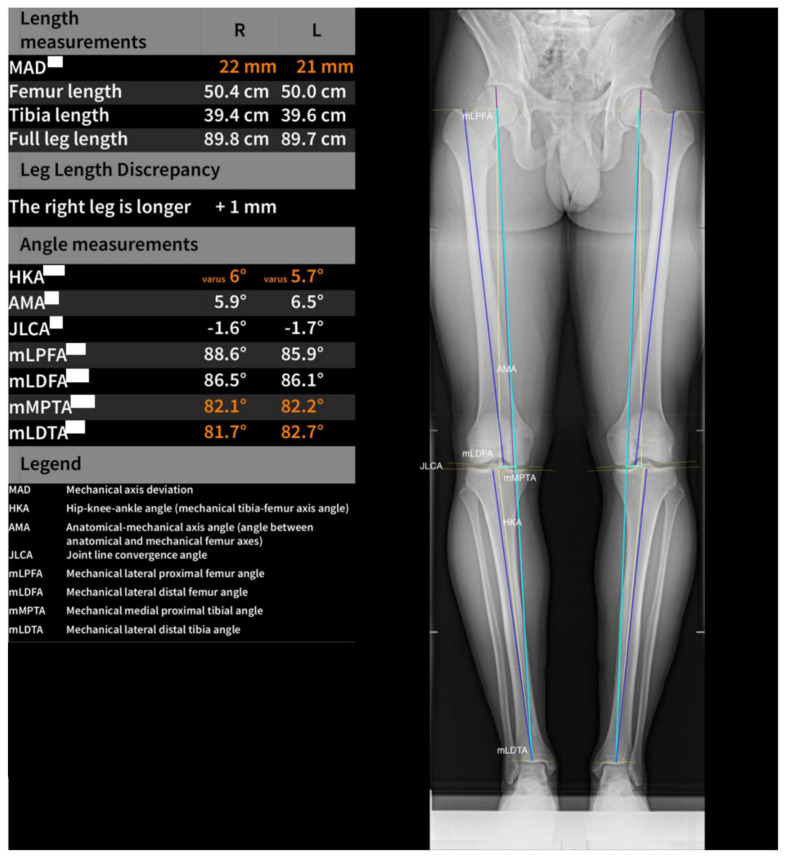
A graphical depiction of software-based measurements of standardized angles on long-leg views of both legs.

The automatic localization of anatomical features of the femur, tibia, and calibration ball to assess all landmarks was needed to perform the required measurements. The AI-based software uses deep learning algorithms and multiple U-Net-based convolutional neural networks. A magnification factor was applied for length measurement based on the detection of a calibration ball. By segmenting a calibration ball and calculating a magnification factor based on the calibration ball size (25 mm) and the diameter of the segmentation (pixel units), the length calibration was performed. The measurement of the following angles was performed on each long-leg radiograph: hip–knee angle (HKA); anatomical–mechanical angle (AMA); joint line convergence angle (JLCA); mechanical lateral distal femoral angle (mLDFA); mechanical lateral distal tibial angle (mLDTA); mechanical lateral proximal femoral angle (mLPFA); mechanical medial proximal tibial angle (mMPTA); mechanical axis deviation (MAD); leg length; femur length; and tibia length. This is further illustrated in Figure 2 and Figure 3.

### 2.3. Statistical Analysis

Statistical analysis was performed using Statistical Package for the Social Sciences (SPSS) version 27.0 software (IBM Corp., Armonk, NY, USA). In order to quantify intra-observer variability as well as variability between manual and computer-aided planning (inter-observer variability), the t-test was used. Paired t-tests were used in order to assess intra-observer variability, while t-tests for independent samples were performed to check for variability regarding manual and computer-aided planning. A confidence interval of 95% was assumed, and a *p*-value < 0.05 was considered statistically significant. Moreover, a descriptive summary of the data was performed using summary tables.

The interrater reliability of measurements was assessed using intraclass correlation coefficients (ICCs) and the confidence interval.

## 3. Results

Axial deviation, femoral and tibial length, and full leg length were measured using the software. At day 1, the axial deviation was a mean of 12.1 cm (SD: 28.6), and the femoral and tibial length was on average 50.4 cm (SD: 3.1cm) and 39.3 cm (SD: 3.0 cm), and full leg length was a mean 89.5 cm (SD: 5.9 cm)At day 15, axial deviation was measured as a mean of 12.4 cm (SD: 28.7 cm), femoral and tibial length were at a mean of 50.4 cm (SD: 3.0 cm) and 39.3 cm (SD: 3.0 cm), and full leg length was measured as 89.6 cm (SD: 5.9 cm) on average.

A descriptive summary of the mean values and standard deviations of the manual and software-based measurements regarding the leg axis angles on days 1 and 15, respectively, is depicted in Table 1.

Concerning intra-observer variability, no statistically significant difference was observed regarding the software-based measurements. However, significant differences were noted concerning intra-observer variability when measuring the mLDFA and AMA manually. Moreover, the other manual measurements showed no statistical significance.

The test for statistical significance regarding the variability between manual and software-based measurements showed different results regarding the individual angles. For some angles, values varied strongly between manual and computer-aided measurements. Statistical significance was detected for mLPFA, mLDFA, mMPTA, and mLPTA on day 1, and mLPFA, mMPTA, and mLPTA on day 15, respectively. A summary of the *p*-values associated with the respective leg axis angles is outlined in Table 2.

The ICC revealed a value of 0.99 for the measurement of the interrater reliability, which, according to [15], corresponds to excellent agreement.

## 4. Discussion

The aim of this prospective study was, therefore, to evaluate the measurement software Image Biopsy Lab (Vienna, Austria) in comparison with conventional manual measurements using inter-observer and intra-observer variability on 2D radiographs.

One of the most important findings of the present study was that regarding four of the seven measured angles on day 1 (mLPFA: *p* = 0.026; mLDFA: *p* = 0.035; mMPTA: *p* < 0.001; mLPTA: *p* < 0.001) and three on day 15 (mLPFA: *p* < 0.001; mMPTA: *p* = 0.010; mLPTA: *p* < 0.001), statistically significant differences between manual and software-based measurements were detected, indicating that manual and software-based planning of leg axes leads to differential results. Additionally, the small difference in inter-observer variability between days 1 and 15 might indicate that the learning curve of performing manual measurements more precisely is quite small after only one repetition. Furthermore, intra-observer variability shows no significant results concerning software-based planning, possibly implying that this method might be more precise, and thus measurements may be more reliable and consistent between different time points. Testing for intra-observer variability of manual measurements revealed statistically significant differences regarding two angles (mLDFA: *p* = 0.012; AMA: *p* = 0.030), pointing towards poorer reliability of manual measurements with increased variability.

Potential explanations for reduced intra-observer variability during software-based analysis include the ability to zoom parts of the radiograph, enabling more exact determination of relevant landmarks for drawing. Moreover, transparent films may slide, which can consequently lead to inaccurate drawings and thus reduced reliability. Additionally, the goniometer has a 1° scale, and no further accuracy is possible.

In 2006, Hankemeier et al. [16] performed an analysis of intra-observer reliability regarding computer-assisted analysis of lower limb geometry and compared these findings to manual measurements on conventional radiographs. In this study, one single surgeon reviewed 59 long-leg radiographs five times and measured the mLPFA, mLDFA, mMPTA, mLDTA, JLCA, and AMA, respectively. The authors concluded that computer-assisted analysis increases intra-observer reliability, which is in accordance with the findings of this present study.

In a more recent study by Schröter et al. [17], the interrater reliability of two digital planning software for high tibial osteotomy was evaluated. In accordance with our results, high interrater reliability could be found using digital planning software.

A similar study reporting on the reliability of an imaging software in the preoperative planning of high tibial osteotomy detected high reliability and consistency between the conventional paper print method and the software-assisted method [18]. This further supports the hypothesis of our study regarding the reliability of measurement software in preoperative planning.

In this study, the anatomical axis was drawn by connecting two central lines through the diaphysis of the femur and tibia. The assessment of the anatomical axis is known to be difficult due to the bowing of the femoral shaft. Moreland [19] defined the anatomical axis as the connecting line between the midpoint of the medial-to-lateral width of the femoral diameter at half of the femoral length and 10 cm above the joint line. The literature showed varying definitions of the anatomical axis, but no significant differences were detected between them [20]. Another problem accompanied with planning on plain radiographs is rotational abnormality. It was shown that planning is rather precise when rotation is neutral, but that pathological rotation of the femur may lead to deviations, making estimated corrections proportional to the degree of malrotation necessary [21].

A further recent study by Pagano et al. [22], evaluating the role and efficiency of AI-powered software in total knee arthroplasty, showed excellent agreement with expert metrics in most knee angles and axial alignments assessed; however, it indicates limitations in the assessment of JLCA, the Mikulicz line, and in patients with a body mass index higher than 30 kg/m^2^, which is comparable to our findings.

Several previous studies have reported on the inter-observer and intra-observer reliability of software-based 2D and 3D planning of component sizes for TKA [23]. It was reported that inter- and intra-observer reliability for component sizes was higher with CT-based 3D planning, comparing directly to two other published research articles that have performed preoperative 2D planning, supporting the fact that 3D planning using CT or MRI may lead to more precise measurements [24,25].

In addition to its use in preoperative planning, artificial intelligence is also used as a diagnostic tool for osteoarthritis of the knee, where studies have shown an increase in interrater reliability, which confirms our findings [26].

There were several limitations associated with the present study. First, measurements were only performed on standardized X-rays that are routinely performed in the preoperative setting. This comes with the advantage that no additional radiation is applied to the patient; however, planning might be more precise when performed on CT imaging, which is, on the other hand, associated with greatly increased radiation exposure compared to conventional radiographs. Second, measurements were only performed by two independent reviewers. The power of the findings might be increased by having the radiographs analyzed by more reviewers and including more experienced specialists or senior physicians. Furthermore, analysis of the radiographs at more than two time points may also enhance the validity and precision of the results. Scale, contrast, and brightness can affect the software evaluation of X-ray images. These factors can affect the software’s ability to recognize landmarks or perform measurements, especially if the contrast quality of the X-ray images is not sufficient or the brightness is not set optimally. To minimize such effects, a standardized acquisition method is used when capturing X-ray images for analysis and to ensure that the image quality is sufficient. Paying attention to these factors and, if necessary, adjusting the settings can help to improve the reliability of software evaluation and the accuracy of manual measurements in orthopaedic imaging. As a university hospital, we are also subject to regular quality controls in order to be able to react accordingly. Additionally, no power analysis for the number of physicians, the number of patient cases, and repetitive measurements was performed. However, [15] postulated that, as a rule of thumb, at least 30 heterogeneous patient cases should be included.

## 5. Conclusions

Preoperative planning of leg axis angles and alignment using planning software showed less inter- and intra-observer variability in contrast to manual measurements, and results differed with respect to manual planning. We believe that the planning software is more reliable and would recommend its use in clinical settings.

## Figures and Tables

**Table 1:** Summary of mean values and standard deviation (SD) at day 1 and day 15.

	Manual d1	Manual d15	Software d1	Software d15
mLPFA	90.3 (SD: 4.7)	89.7 (SD: 3.9)	91.7 (SD: 4.8)	91.8 (SD: 4.8)
mLDFA	89.2 (SD: 4.1)	88.0 (SD: 3.9)	88.1 (SD: 2.9)	88.2 (SD: 2.9)
JLCA	3.3 (SD: 3.7)	3.3 (SD: 3.9)	2.4 (SD: 4.0)	2.5 (SD: 4.0)
mMPTA	89.5 (SD: 3.3)	88.9 (SD: 3.4)	87.8 (SD: 2.8)	87.8 (SD: 2.8)
mLPTA	88.5 (SD: 3.5)	87.9 (SD: 3.2)	86.2 (SD: 3.8)	86.1 (SD: 3.8)
HKA	2.6 (SD: 7.1)	2.8 (SD: 7.6)	2.6 (SD: 7.6)	2.7 (SD: 7.6)
AMA	6.7 (SD: 1.1)	7.0 (SD: 1.6)	6.9 (SD: 1.1)	6.9 (SD: 1.1)

Values are in degrees; SD = standard deviation; d1 = day 1; d15 = day 15; manual = manual measurements of both readers.

**Table 2:** Summary of intra- and inter-observer variability.

Knee Angles	Intra-Observer Variability (Manual)	Intra-Observer Variability (Software)	Inter-Observer Variability d1	Inter-Observer Variability d15
mLPFA	*p* = 0.285	*p* = 0.320	*p* = 0.026	*p* < 0.001
mLDFA	*p* = 0.012	*p* = 0.320	*p* = 0.035	*p* = 0.741
JLCA	*p* = 0.822	*p* = 0.435	*p* = 0.114	*p* = 0.103
mMPTA	*p* = 0.164	*p* = 0.320	*p* < 0.001	*p* = 0.010
mLPTA	*p* = 0.097	*p* = 0.276	*p* < 0.001	*p* < 0.001
HKA	*p* = 0.726	*p* = 0.320	*p* = 0.934	*p* = 0.943
AMA	*p* = 0.030	*p* = 0.320	*p* = 0.087	*p* = 0.594

d1 = day 1, d15 = day 15.

## Data Availability

The data presented in this study are available on request from the corresponding author.

## References

[1] Berend M.E., Small S.R., Ritter M.A., Buckley C.A., Merk J.C., Dierking W.K. (2010). Effects of femoral component size on proximal tibial strain with anatomic graduated components total knee arthroplasty. J. Arthroplast..

[2] Ritter M.A., Faris P.M., Keating E.M., Meding J.B. (1994). Postoperative alignment of total knee replacement. Its effect on survival. Clin. Orthop. Relat. Res..

[3] Kinzel V., Scaddan M., Bradley B., Shakespeare D. (2004). Varus/valgus alignment of the femur in total knee arthroplasty. Can accuracy be improved by pre-operative CT scanning?. Knee.

[4] Kniesel B., Konstantinidis L., Hirschmüller A., Südkamp N., Helwig P. (2014). Digital templating in total knee and hip replacement: An analysis of planning accuracy. Int. Orthop..

[5] Schroer W.C., Berend K.R., Lombardi A.V., Barnes C.L., Bolognesi M.P., Berend M.E., Ritter M.A., Nunley R.M. (2013). Why are total knees failing today? Etiology of total knee revision in 2010 and 2011. J. Arthroplast..

[6] Goyal N., Stulberg S.D. (2015). Evaluating the Precision of Preoperative Planning in Patient Specific Instrumentation: Can a Single MRI Yield Different Preoperative Plans?. J. Arthroplast..

[7] Hirschmann M.T., Konala P., Amsler F., Iranpour F., Friederich N.F., Cobb J.P. (2011). The position and orientation of total knee replacement components: A comparison of conventional radiographs, transverse 2D-CT slices and 3D-CT reconstruction. J. Bone Jt. Surg. Br..

[8] Tiefenboeck S., Sesselmann S., Taylor D., Forst R., Seehaus F. (2022). Preoperative planning of total knee arthroplasty: Reliability of axial alignment using a three-dimensional planning approach. Acta Radiol..

[9] The B., Diercks R.L., van Ooijen P.M.A., van Horn J.R. (2005). Comparison of analog and digital preoperative planning in total hip and knee arthroplasties. A prospective study of 173 hips and 65 total knees. Acta Orthop..

[10] Hsu A.R., Kim J.D., Bhatia S., Levine B.R. (2012). Effect of training level on accuracy of digital templating in primary total hip and knee arthroplasty. Orthopedics.

[11] Kobayashi A., Ishii Y., Takeda M., Noguchi H., Higuchi H., Toyabe S. (2012). Comparison of analog 2D and digital 3D preoperative templating for predicting implant size in total knee arthroplasty. Comput. Aided Surg..

[12] Ettinger M., Claassen L., Paes P., Calliess T. (2016). 2D versus 3D templating in total knee arthroplasty. Knee.

[13] Jazrawi L.M., Birdzell L., Kummer F.J., Di Cesare P.E. (2000). The accuracy of computed tomography for determining femoral and tibial total knee arthroplasty component rotation. J. Arthroplast..

[14] Konigsberg B., Hess R., Hartman C., Smith L., Garvin K.L. (2014). Inter- and intraobserver reliability of two-dimensional CT scan for total knee arthroplasty component malrotation. Clin. Orthop. Relat. Res..

[15] Koo T.K., Li M.Y. (2016). A Guideline of Selecting and Reporting Intraclass Correlation Coefficients for Reliability Research. J. Chiropr. Med..

[16] Hankemeier S., Gosling T., Richter M., Hufner T., Hochhausen C., Krettek C. (2006). Computer-assisted analysis of lower limb geometry: Higher intraobserver reliability compared to conventional method. Comput. Aided Surg..

[17] Schröter S., Ihle C., Mueller J., Lobenhoffer P., Stöckle U., van Heerwaarden R. (2013). Digital planning of high tibial osteotomy. Interrater reliability by using two different software. Knee Surg. Sports Traumatol. Arthrosc..

[18] Lee Y.S., Kim M.K., Byun H.W., Kim S.B., Kim J.G. (2015). Reliability of the imaging software in the preoperative planning of the open-wedge high tibial osteotomy. Knee Surg. Sports Traumatol. Arthrosc..

[19] Moreland J.R. (1988). Mechanisms of failure in total knee arthroplasty. Clin. Orthop. Relat. Res..

[20] Gopurathingal A.A., Bhonsle S. (2021). Inter-Observer and Intra-Observer Reliability of 2D Radiograph-Based Valgus Cut Angle Measurement in Preoperative Planning for Primary Total Knee Arthroplasty. Cureus.

[21] Swanson K.E., Stocks G.W., Warren P.D., Hazel M.R., Janssen H.F. (2000). Does axial limb rotation affect the alignment measurements in deformed limbs?. Clin. Orthop. Relat. Res..

[22] Pagano S., Müller K., Götz J., Reinhard J., Schindler M., Grifka J., Maderbacher G. (2023). The Role and Efficiency of an AI-Powered Software in the Evaluation of Lower Limb Radiographs before and after Total Knee Arthroplasty. J. Clin. Med..

[23] Miura M., Hagiwara S., Nakamura J., Wako Y., Kawarai Y., Ohtori S. (2018). Interobserver and Intraobserver Reliability of Computed Tomography-Based Three-Dimensional Preoperative Planning for Primary Total Knee Arthroplasty. J. Arthroplast..

[24] Kastner N., Aigner B.A., Meikl T., Friesenbichler J., Wolf M., Glehr M., Gruber G., Leithner A., Sadoghi P. (2014). Gender-specific outcome after implantation of low-contact-stress mobile-bearing total knee arthroplasty with a minimum follow-up of ten years. Int. Orthop..

[25] Kastner N., Sternbauer S., Friesenbichler J., Vielgut I., Wolf M., Glehr M., Leithner A., Sadoghi P. (2014). Impact of the tibial slope on range of motion after low-contact-stress, mobile-bearing, total knee arthroplasty. Int. Orthop..

[26] Neubauer M., Moser L., Neugebauer J., Raudner M., Wondrasch B., Führer M., Emprechtinger R., Dammerer D., Ljuhar R., Salzlechner C. (2023). Artificial-Intelligence-Aided Radiographic Diagnostic of Knee Osteoarthritis Leads to a Higher Association of Clinical Findings with Diagnostic Ratings. J. Clin. Med..

